# Prostaglandin E2 produced following infection with Theiler's virus promotes the pathogenesis of demyelinating disease

**DOI:** 10.1371/journal.pone.0176406

**Published:** 2017-04-26

**Authors:** Seung Jae Kim, Young-Hee Jin, Byung S. Kim

**Affiliations:** Department of Microbiology-Immunology, Northwestern University Medical School, Chicago, Illinois; University of the Pacific, UNITED STATES

## Abstract

Infection of various cells with Theiler’s murine encephalomyelitis virus (TMEV) activates the TLR- and melanoma differentiation-associated gene 5 (MDA5)-dependent pathways, resulting in the production of IL-1β via the activation of caspase-1 upon assembly of the node-like receptor protein 3 (NLRP3) inflammasome. The role of IL-1β in the pathogenesis of TMEV-induced demyelinating disease was previously investigated. However, the signaling effects of prostaglandin E2 (PGE_2_) downstream of the NLRP3 inflammasome on the immune responses to viral determinants and the pathogenesis of demyelinating disease are unknown. In this study, we investigated the levels of intermediate molecules leading to PGE_2_ signaling and the effects of blocking PGE_2_ signaling on the immune response to TMEV infection, viral persistence and the development of demyelinating disease. We demonstrate here that TMEV infection activates the NLRP3 inflammasome and PGE_2_ signaling much more vigorously in dendritic cells (DCs) and CD11b^+^ cells from susceptible SJL mice than in cells from resistant B6 mice. Inhibition of virus-induced PGE_2_ signaling using AH23848 resulted in decreased pathogenesis of demyelinating disease and viral loads in the central nervous system (CNS). In addition, AH23848 treatment caused the elevation of protective early IFN-γ-producing CD4^+^ and CD8^+^ T cell responses. Because the levels of IFN-β were lower in AH23848-treated mice but the level of IL-6 was similar, over-production of pathogenic IFN-β was modulated and the generation of IFN-γ-producing T cell responses was enhanced by the inhibition of PGE_2_ signaling. These results strongly suggest that excessive activation of the NLRP3 inflammasome and downstream PGE_2_ signaling contribute to the pathogenesis of TMEV-induced demyelinating disease.

## Introduction

Infection of various cells with Theiler’s murine encephalomyelitis virus (TMEV) activates the production of various cytokines via toll like receptor (TLR)- and melanoma differentiation-associated gene 5 (MDA5)-dependent pathways [1–3]. TLR-mediated signaling results in the polymerization of the node-like receptor protein 3 (NLRP3) inflammasome, leading to the activation of caspase-1 and the subsequent production of the proinflammatory cytokines IL-1β and IL-18 [4–6]. In addition, MDA-5 signaling cooperatively promotes the activation of NLRP3 [7, 8]. We have previously demonstrated that the balance of IL-1 signaling is important for the pathogenesis of TMEV-induced demyelinating disease [9]. The presence of high IL-1 levels exerts a pathogenic role by elevating pathogenic Th17 responses, whereas the lack of IL-1 signals promotes viral persistence in the spinal cord due to insufficient T cell activation.

The above observations strongly suggest that the activation levels of NLRP3 by viral infection may strongly affect the protection or pathogenesis of the host via the differences in the downstream cytokine production. Although the cause of multiple sclerosis (MS) is unknown, one or multiple infectious agents may be involved in the initial infliction of tissue damage leading to the autoimmunity [10–13]. TMEV infection leads to the development of chronic demyelinating disease in susceptible mice and this virus-induced demyelinating disease is considered as a relevant model of MS [14, 15]. However, the direct involvement of the NLRP3 inflammasome in the activation of caspase-1, leading to the production of IL-1β following TMEV infection, was not investigated. Furthermore, the downstream mechanisms associated with the effects of IL-1β on the pathogenesis of virally induced demyelinating disease remain unknown. These downstream cytokines are also associated with MS. Therefore, the activation levels of NLRP3 upon viral infection are likely to affect the pathogenesis and these results may also help to understand the pathogenesis of MS.

Proinflammatory stimuli such as IL-1β, which are downstream products of NLRP3 activation, induce the production of cyclooxygenase 2 (COX-2) and membrane-bound prostaglandin E synthesis-1 (mPGES-1), which participate in the production of prostaglandin E_2_ (PGE_2_) [16–18]. COX-2 converts arachidonic acid to prostaglandin G_2_ (PGG_2_) intermediate, which is subsequently converted to PGE_2_ by mPGES-1. In addition to the inducible forms (COX-2 and mPGES-1), constitutive forms of COX-1 and microsomal PGES-2 (mPGES-2) also participate in the generation of PGE_2_ [17, 19, 20]. Many different viral infections closely related to TMEV, including Coxsackie virus B3, are known to induce COX-2 and/or PGE_2_, which affect viral pathogenesis [21]. In addition, it has previously been shown that COX-2 expression is upregulated on oligodendrocytes of TMEV-infected mice, and treatment of the mice with COX-2 inhibitors limits the development of TMEV-induced demyelinating disease [22]. These observations strongly suggest that COX-2 and/or its downstream signaling pathways are likely involved in the pathogenesis of TMEV-induced demyelinating disease and of other many other inflammatory viral diseases.

PGE_2_ is an important inflammatory mediator in response to cellular stimuli and is a key mediator in chronic inflammatory disease and cancer [23]. PGE_2_ functions through its receptors EP1-EP4. Among the receptors of PGE_2_, EP4 on T cells and dendritic cells facilitates Th1 cell differentiation and Th17 cell expansion [24]. In addition, PGE_2_ regulates Th17 cell differentiation and function. Additionally, the presence of PGE_2_ during T cell stimulation surges the production of IL-17 but reduces IFN-γ production [25]. Furthermore, PGE_2_ suppresses the effector functions of macrophages, Th1, CTLs, and natural killer cells, but it promotes Th2, Th17, and regulatory T cell responses [23]. Th1 and Th17 responses to viral epitopes play a critical role in the development of TMEV-induced demyelinating disease [26, 27]. Furthermore, TMEV infection induces strong TLR and MDA5 signals, which affect the level and type of immune responses and generate PGE_2_. Many different bacterial and viral infections ultimately induce PGE_2_ via the activation of various toll-like receptors (TLRs) and TNF-α [20, 28].

In this study, we assessed the levels of PGE_2_ and its intermediate components after TMEV infection of various glial cells and DCs, which are integral parts of the TMEV-induced demyelinating disease process [29, 30]. In addition, we determined the effects of blocking PGE_2_ signaling on the TMEV-specific immune response, viral persistence and the development of demyelinating disease. TMEV infection activated the NLRP3 inflammasome and PGE_2_ signaling (COX-2 and PGES-1) much more vigorously in DCs and brain cells, including primary microglia, oligodendrocytes and astrocytes, from susceptible SJL mice compared to cells from resistant B6 mice. Blocking PGE_2_ signaling using AH23848 [31, 32] resulted in decreased pathogenesis of TMEV-induced demyelinating disease and decreased viral loads in the CNS. The treatment enhanced the development of protective early IFN-γ-producing CD4^+^ and CD8^+^ T cell responses. However, the level of IFN-β was lower in AH23848-treated mice, but the level of IL-6 was similar between the two groups. These results strongly suggest that excessive activation of the NLRP3 inflammasome and the resulting downstream PGE_2_ signaling significantly contribute to the pathogenesis of TMEV-induced demyelinating disease. Therefore, blocking PGE_2_ signaling would provide a powerful means to control virus-induced chronic inflammatory diseases and other inflammatory diseases.

## Materials and methods

### Mice

C57BL/6 and SJL mice were purchased from Harlen Laboratories (Indianapolis, IN, USA). All experiments were conducted with 6- to 10-week-old females. Experimental procedures with mice were conducted in accordance with the NIH animal care guidelines and were approved by the animal care and use committee of Northwestern University (#2011–1316). Mice were anesthetized with isoflurane in the hood and then sacrificed by cervical dislocation. Virus infected mice were anesthetized with isoflurane, followed by thorocotomy. If further maintenance of virus infected, severely impaired mice were required, moistened food pellets, water bottles and/or nutricals were placed on the floor of the cages. Mice were kept in an environmental temperature between 65-75°F and the humidity levels between 40% and 60% at a 14-hour light/10-hour dark cycle.

### Synthetic peptides

All synthetic peptides were purchased from Genmed Synthesis (San Francisco, CA). Stock peptides of the previously defined TMEV-specific CD4^+^ (VP2_203-220_ and VP4_25-38_) and CD8^+^ T cell epitopes (VP2_121-130_ and VP3_110-120_) for SJL mice were prepared in 8% dimethylsulfoxide in phosphate buffered saline (PBS).

### Viral infection

For in vitro viral infection, cells were washed and subsequently incubated in infection media (DMEM supplemented with 0.1% bovine serum albumin) with the BeAn strain of TMEV at various multiplicity of infection (MOI). For viral infection in mice, approximately 30 μl (1x10^6^ PFU) of TMEV was injected into the right hemisphere of 5- to 7-week-old SJL mice anesthetized with isoflurane as previously described [14, 33].

### Reagent and mouse inoculation

AH23848, the PGE_2_ receptor 4 (EP4) antagonist, was purchased from Cayman Chemical (Ann Arbor, Michigan, USA). AH23848 was dissolved in DMSO (5 mg/ml [1 mg vial /200 μl DMSO]) and further diluted in DMEM media (10-fold [200 μl stock in DMSO/1800 μl of media]). AH23848 (5 mg/kg, approximately 100 μg/each mouse [200 μl of diluted solution was inoculated]) or DMSO vehicle control (200 μl DMSO/1800 μl media) was injected intraperitoneally at 1, 5, and 8 dpi. To block early effects of virus-induced activation of EP4 signaling on the development of viral demyelination, only minimal amount and frequency of the treatment with EP4 receptor antagonist were used, similar to the treatment previously described [24, 31, 32, 34].

### Generation of bone-marrow (BM) DCs

BM cells were harvested from femurs and tibias of SJL or B6 mice and cultured in RPMI-10 with 20 ng/ml murine rGM-CSF (PeproTech) as described [35]. On day 5, predominantly immature DCs were obtained. For DC expansion experiments, BMCs cultured for 3 d were collected and used.

### Primary glial cell cultures

Primary glial cells were derived from 0- to 3-day-old neonatal SJL and C57BL/6 mice by using differential shaking [36]. Single cell suspensions from neonatal brains were seeded on poly-L-lysine-coated flasks (25 μg/ml), in DMEM supplemented with 2 mM L-glutamine, antibiotics (Gibco BRL, Grand Island, NY), and 10% fetal calf serum (<10 U/ml Endotoxin, HyClone, Logan, UT, USA). After 8 days in culture at 37°C, flasks were placed in a shaker at 200 rpm overnight and attached cells were cultured further for another 4 days at 37°C [37]. Detached oligodendrocyte/microglia mixture cells were removed by shaking at 200 rpm for 1 h. At 2 h after seeding, gentle aspirates of the old media contaminating oligodendrocytes were counted using hemocytometer and re-seeded 1 X 10^5^ cells per well in coated 6 well-plates. The firmly attached microglia were resuspended in fresh media for 2 h and harvested after trypsinization. Cells were then resuspended in fresh medium, counted and reseeded in 6 well-plates as above. Cells remain attached in the original cultures were incubated additionally for 4 d and then agitated overnight (250 rpm) to harvest adherent astrocytes. After 8 days in culture at 37°C, flasks were placed in a shaker at 200 rpm overnight and attached cells were cultured further for another 4 days at 37°C. After removing detached microglial cells by shaking at 200 rpm for 1h, cultures were incubated for 4 d and then agitated overnight (250 rpm) to harvest adherent astrocytes. The cell preparations were at least 90% pure, as confirmed by staining with antibodies specific for the microglial, oligodendrocyte and astrocyte markers Mac-1 (Boehringer Mannheim, Indianapolis, IN, USA), CNPase (Sigma-Aldrich, St. Louis, MO, USA) and GFAP (Dako, Carpinteria, CA, USA), respectively.

### Assessment of TMEV-induced demyelinating disease

Clinical symptoms of disease in TMEV-infected mice were assessed weekly, as previously described [38]. The clinical score was determined based on the following scale: grade 0, no clinical signs; grade 1, mild waddling gait; grade 2, moderate waddling gait and hind-limb paresis; grade 3, severe hind-limb paralysis; grade 4, severe hind-limb paralysis and loss of righting reflex; and grade 5, moribund or death.

### Histopathology

Mice at 43 days post-infection were perfused with 50 mL of PBS via intracardiac puncture. Brains and spinal cords from SJL mice and PEG_2_ antagonist-treated SJL mice were dissected and fixed in 4% formalin in PBS for 24 h, embedded in OCT and stored at -70°C until sectioning and staining. The brains and spinal cords were sectioned at 6 μm and evaluated by hematoxylin and eosin (H&E) staining for inflammatory infiltrates, Luxol Fast Blue (LFB) staining for axonal demyelination, and Bielschowsky silver staining for axon loss and damage. Four different sections of the cerebellum of the brain and the lumbar region of the spinal cords per experimental group were examined using a Leica DMR fluorescent microscope, and images were captured using an AxioCam MRc camera and AxioVision imaging software.

### RT-PCR and real-time PCR

Total RNA was purified from the lysates of brain/spinal cord cells using TRIzol Reagent (Invitrogen, Carlsbad, CA, USA) according to the manufacturer’s instructions. First-strand cDNA was synthesized by MMLV reverse transcriptase and oligo (dT)_18_ from 1–4 μg total RNA depending on the frequencies of the messages. The MJ Research, Inc. (Watertown, MA, USA) thermal cycler was used for PCR. Primers were obtained from Integrated DNA Technologies (Coralville, IA, USA). The sense and antisense primer sequences are as follows: prostaglandin-endoperoxide synthase 2 (Ptgs2), Cox2 (5’-AACCTCGTCCAGATGC-TATCTTTGGG-3’ and 5’-GTGCTCGGCTTCCAGTATTGAGGA-3’); prostaglandin E synthase (Ptges) (5’-GGCCCAGTTCTCCTGTTTCTCCAT-3’ and 5’-AGAGACACCAAGTCCGCAAGTTCA-3’); NLRP3 (5’-TGGGCAACAATGATCTTGGCGA-3’ and 5’-GGAGCGCTTCTAAGGCACGTTT-3’); IL-6 (5’-AGTTGCCTTCTTGGGACTGA-3’ and 5’-TCCACGATTTCCCAGAGAAC-3’); IFN-α4 (5’-AAGGCTCAAGCCATCCTTGTGCTA-3’ and 5’-TTGCCAGCAAGTTGGTGAGGAAG-3’); IFN-β (5’-CCCTATGGAGATGACG-GAGA-3’ and 5’-CTGTCTGCTGGTGGAGTTGA-3’); IFN-γ (5’-ACTGGCAAAAGGA-TGGTGAC-3’ and 5’-TGAGCTCATTGAATGCTTGG-3’); GAPDH (5’-AACTTTGGC-ATTGTGGAAGG-3’ and 5’-ACACATTGGGGGTAGGAACA-3’); and TMEV genome (5’-CCCAGTCCTCAGGAAATGAAGG-3’ and 5’-TCCAAAAGGAGAGGTGCCATAG-3’). For quantitative analysis of gene expression, real-time PCR was performed using iCycler SYBR green I mastermix and an iCycler real-time machine (Bio-Rad, Hercules, CA, USA). GAPDH expression served as an internal reference for normalization of each cDNA. The expression level represents fold-increase compared to the lowest values in the group. Every real-time PCR reaction was performed in triplicate.

### ELISA

Cytokine levels produced by T cells stimulated with isolated APCs were determined after a 36-hr incubation. IFN-γ levels were assessed using ELISA (OPTEIA kit; BD Pharmingen, San Diego, CA, USA). The plates were read using a Spectra MAX 190 microplate reader (Molecular Devices, Sunnyvale, CA, USA) at a 450 nm wavelength.

### Intracellular cytokine staining

Brains and spinal cords were removed from mice after perfusion with Hank’s balanced salt solution (HBSS) through the left ventricle. The tissues were forced through steel mesh to prepare single-cell suspensions and incubated at 37° C for 45 min in 250 μg/ml collagenase type 4 (Worthington Biochemical Corp., Lakewood, NJ, USA). A continuous 100% Percoll gradient (Pharmacia, Piscataway, NJ, USA) was constructed at 27,000 x g for 30 min to enrich for CNS-infiltrating mononuclear cells. CNS mononuclear cells from mice were cultured in plates coated with either PMA plus ionomycine or viral epitope peptides in the presence of Golgi-Plug for 6 h at 37°C. The cells were then incubated in 50 μl of 2.4G2 hybridoma (American Type Culture Collection, Manassas, VA, USA) supernatant for 30 min at 4°C to block the Fc receptors. The cells were incubated for an additional 30 min at 4°C in the presence of allophycocyanin-conjugated anti-CD8 (clone 53–6.7) or anti-CD4 (GK1.5) antibodies diluted in 50 μl of Fc-block (2.4G2 supernatant). After two washes, intracellular IFN-γ staining was performed according to the manufacturer's instructions (BD Pharmingen, San Diego, CA, USA) using phycoerythrin-labeled rat monoclonal anti-IFN-γ antibody (XMG1.2). The cells were analyzed on a Becton Dickinson FACSCalibur flow cytometer.

### Statistical analyses

The significance of the difference (two-tailed p value) between the experimental group with various treatments and the control group was analyzed with an unpaired Student’s t-test, otherwise indicated, using InStat Program (GraphPAD Software, San Diego, CA, USA). Multiple group comparisons were done by a one-way analysis of variance (ANOVA) with Tukey-Kramer post-hoc analysis. Values of *p*<0.05 were considered significant.

## Results

### Elevated induction of the NLRP3 inflammasome after TMEV infection in DCs and glial cells from SJL mice compared to cells from B6 mice

**Fig 1:**
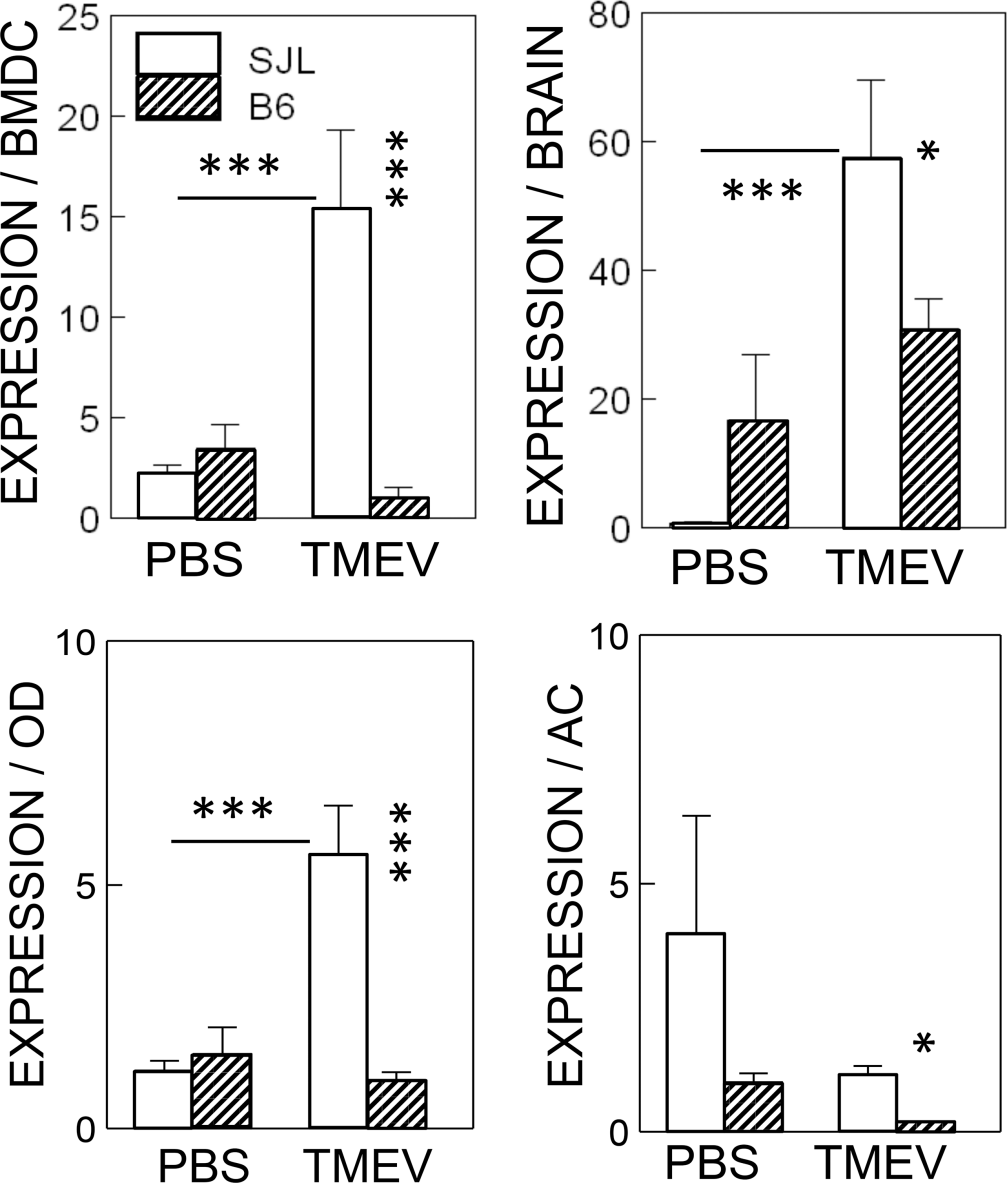
Levels of NLRP3 inflammasome activation after infection with TMEV in various cells from susceptible SJL and resistant B6 mice. Bone marrow-derived dendritic cells (BMDCs), neonatal whole brain cells, and glial cells (OD, oligodendrocytes and AS, astrocytes) derived from neonatal brains of SJL and B6 mice were compared after infection with or without TMEV for 24 h. Multiple group comparisons were done by a one-way analysis of variance (ANOVA) with Tukey-Kramer post-hoc analysis. *, P < 0.05; ***, p < 0.001. The error bars represent standard deviations (SD) of the triplicate samples.

We have previously demonstrated that the level of IL-1β plays a pivotal role in the pathogenesis of TMEV-induced demyelinating disease as well as in protection from the disease [9]. To determine the involvement of the upstream and the downstream signals of IL-1β, we assessed the expression levels of the upstream NLRP3 inflammasome in dendritic cells and different glial cells from resistant B6 and susceptible SJL mice after TMEV infection (Fig 1). Significant upregulation of NLRP3 expression (*p*<0.001) was observed in all cell types (bone marrow-derived dendritic cells, neonatal brain cells, and neonatal oligodendrocytes), but not in neonatal astrocytes from susceptible SJL mice, after infection (MOI = 10) with TMEV. In contrast, no significant upregulation of NLRP3 (*p*>0.05) was observed in these cell types from resistant B6 mice. Consequently, the levels of NLRP3 in all the cell types from SJL mice after TMEV infection were significantly higher (*p*<0.05) than in those from infected B6 mice. It is most likely that the NLRP3 inflammasome is activated via TLR3, TLR2 and/or MDA5 upon TMEV infection [1–3].

### Higher levels of PGE_2_ signaling components in glial cells from SJL mice compared to B6 mice after TMEV infection

**Fig 2:**
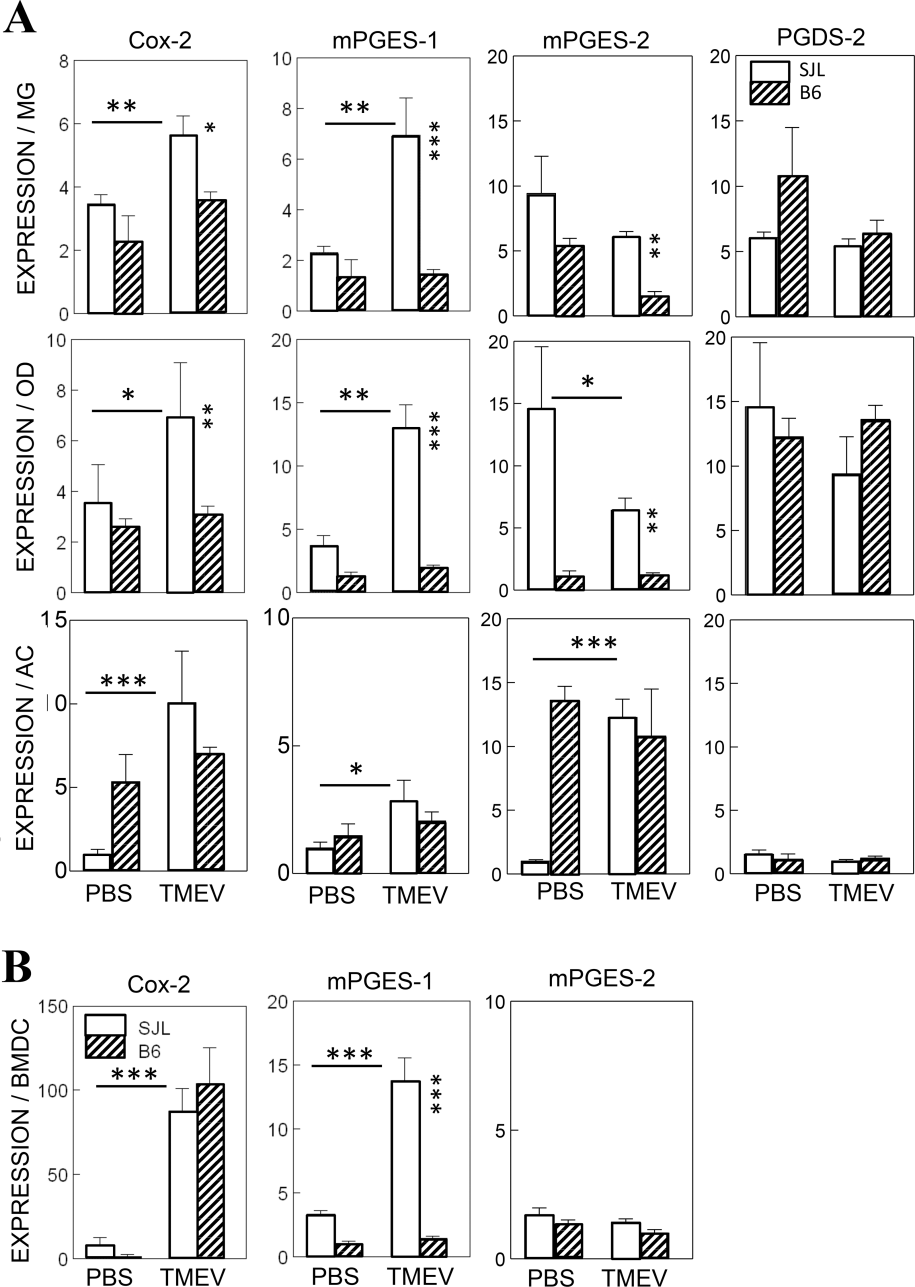
Elevation of COX-2 and mPGES-1 levels in various cells infected with TMEV. **A.** The levels of inducible COX-2 and mPGES-1, constitutive mPGES-2 and antagonistic PGDS- 2 in primary microglia, oligodendrocytes and astrocytes derived from B6 or SJL mice were assessed using qPCR after infection with TMEV (MOI = 10) for 24 h. **B.** The levels of Cox-2, mPGES-1 and mPGES-2 in bone marrow-derived dendritic cells (BMDCs) from resistant B6 and susceptible SJL mice were assessed using qPCR after infection with TMEV (MOI = 10) for 24 h. MOI represents the multiplicity of infection. For the cells derived from SJL mice, 2 μg of RNA and 4 μg of RNA were used for cells derived from B6 mice due to the relative levels of specific mRNAs. The error bars represent standard deviations (SD) of the triplicate samples. Multiple group comparisons were done by a one-way analysis of variance (ANOVA) with Tukey-Kramer post-hoc analysis. *, P < 0.05; **, P < 0.01; ***, p < 0.001.

To further investigate the potential involvement of PGE_2_, which is a downstream product of IL-1β signaling, in the pathogenesis of TMEV-induced demyelinating disease, the levels of inducible COX-2 and PGES-1 mRNAs in DCs and glial cells (microglia, oligodendrocytes and astrocytes) were assessed after TMEV infection (Fig 2A). The expression levels of COX-2 mRNA in TMEV-infected glial cell types from SJL mice (microglia, *p*<0.01; oligodendrocytes, p<0.05; and astrocytes, *p*<0.001) were significantly higher than those in uninfected cells. However, the levels of COX-2 mRNA were not elevated in these glial cell types from B6 mice after TMEV infection. In contrast, the levels of COX-2 mRNA in infected DCs from SJL and B6 mice were both similarly (*p*<0.001) elevated (Fig 2B). The significant induction of the PGE_2_-specific synthase mPGES-1 (*p*<0.05) was observed only in cells from SJL mice. However, no significant induction of mPGES-1 was observed in any of the cell types tested from B6 mice, including bone marrow derived dendritic cells (BMDCs). These results clearly demonstrate that after TMEV infection, the expression levels of the two major components to generate PGE_2_ signaling, COX-2 and mPGES-1, are significantly higher (*p*<0.05) in cells from susceptible mice but not in those from resistant mice.

In addition to the inducible components of PGE_2_, levels of a constitutive component of PGE2 (mPGES-2) and the opposing component, PGDS-2, were also assessed after TMEV infection to determine possible alterations in these components due to virus-induced cell death or unknown stimulation (Fig 2). These PGE_2_-associated signals are expressed in some brain cells, including microglia [39, 40]. Interestingly, there was no consistent pattern of expression among the glial cell types (Fig 2A). TMEV-infected microglia and oligodendrocytes displayed similar or slightly reduced levels of mPGES-2 mRNA in both SJL and B6 cells. On the other hand, TMEV-infected astrocytes from SJL mice were significantly elevated (*p*<0.001), while infected B6 astrocytes had unchanged high levels in both infected or uninfected cells. There were virtually no changes and no differences in mPGES-2 expression between infected or uninfected BMDCs from SJL and B6 mice (Fig 2B). These results indicate that the constitutive component for generating PGE_2_ varies significantly depending on the cell type and that the variation is particularly great in cell types from susceptible SJL mice. The levels of antagonistic PGDS-2 expression were similar in infected or uninfected glial cells from SJL and B6 mice.

### Inhibition of pathogenesis of TMEV-IDD with blockade of PGE_2_ receptor (EP_4_) signaling in susceptible SJL mice

**Fig 3:**
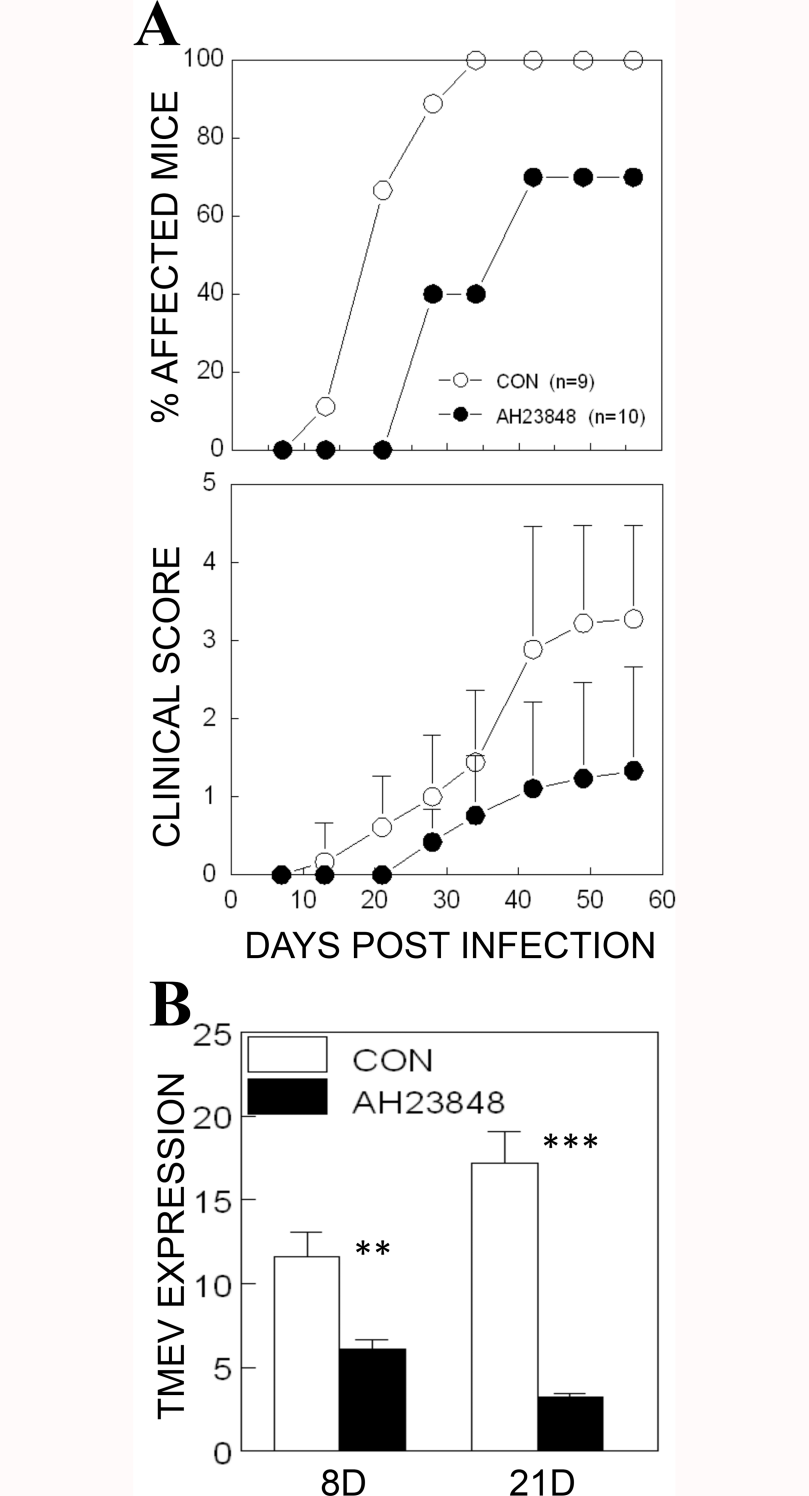
Inhibition of the pathogenesis of demyelinating disease and viral loads in mice treated with a PGE_2_ inhibitor, AH23848. **A.** TMEV was inoculated by intracerebral injection (1x10^6^ PFU) into AH23848-treated SJL mice (n = 10) and DMSO vehicle-treated control SJL mice (n = 9) at 1, 5, and 8 dpi. The percentage of affected mice and the mean clinical scores (+/- SD) were monitored weekly. Clinical scores of individual mice were shown in S1 Table. The two-tailed *p* value between the control and AH23848-treated groups was very significant (*p* < 0.0059) based on the paired t test of the mean clinical cores (t = 4.582 with 5 degrees of freedom) between days 21 and 56 post-infection. **B.** Viral load levels in the CNS of AH23848-treated and control mice were assessed at 8 and 21 dpi using qPCR. The significance of the difference was assessed using two-tailed unpaired Student t test. **, P < 0.01; ***, P < 0.001.

The fact that deletion of COX-2 or its EP_4_ receptor leads to a significant delay in the onset of EAE indicates that PGE_2_ signaling plays an important role in the pathogenesis of demyelinating disease [41, 42]. In addition to the genetic deletion, pharmacological agents such as AH23848 were also successfully used previously to inhibit COX-2/EP_4_ receptor signaling [43]. In this study, we intraperitoneally administered (5 mg/Kg) either AH23848 or vehicle control at 1, 5 and 8 days post-infection (dpi), and then disease development was monitored weekly (Fig 3A). The severity and incidence of disease development were significantly lower in AH23848-treated mice compared to the control mice after 21 dpi (*p*< 0.006 based on the paired t test). These results strongly suggest that COX-2/EP_4_-dependent PGE_2_ signaling is also critical for the development of TMEV-induced demyelinating disease, similar to the development of EAE.

To correlate the disease development patterns with the levels of viral loads in the CNS, we assessed the TMEV RNA levels in the brains and spinal cords using quantitative PCR (Fig 3B). The levels of viral loads in the CNS of AH23848-treated SJL mice were significantly lower at 8 and 21 dpi (*p*<0.01 and *p*<0.001, respectively) compared to the vehicle-treated control mice. These results indicate that PGE_2_ signaling following TMEV infection contributes to the viral loads in the CNS and thereby promotes the pathogenesis of demyelinating disease.

**Fig 4:**
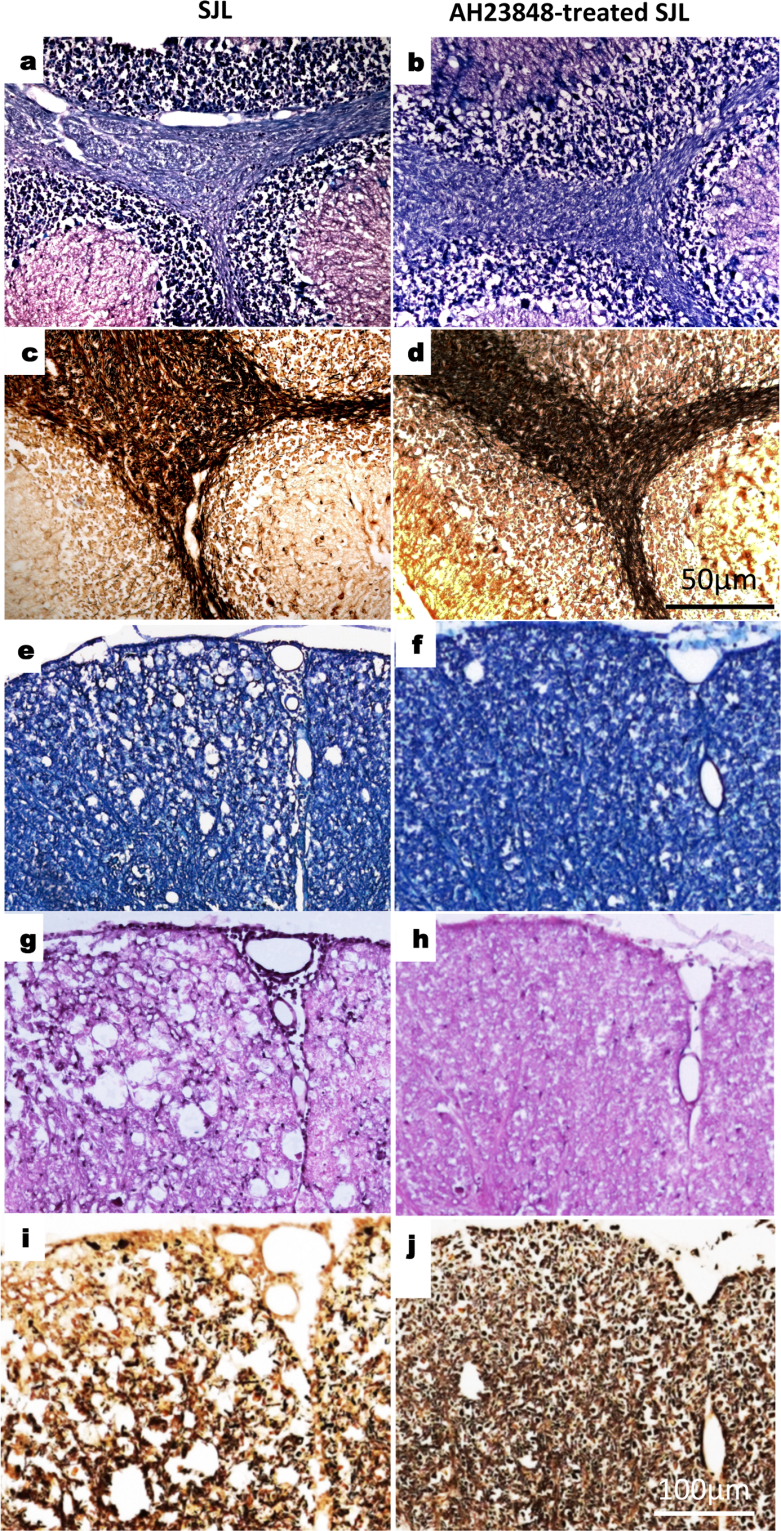
Reduced histopathology in TMEV-infected mice treated with AH23848. Four different sections of the brain and spinal cords of individual mice of two to three per experimental group were graded for demyelination, mononuclear infiltration, and inflammation. A representative sample is shown. Luxol Fast Blue (LFB) staining and counterstaining with H&E showed irregular vacuolation and demyelination in the white matter of the cerebellum of SJL mice (a) and reduced demyelination in PEG_2_ antagonist AH23848-treated SJL mice (b). Luxol Fast Blue (LFB) staining showed irregular vacuolation and demyelination in the white matter of spinal cords of SJL mice (e) and reduced demyelination in A23848-treated SJL mice (f). By H&E staining, meningitis was evident in the spinal cord of control SJL mice (g) and was not observed in A23848-treated SJL mice (h). Bielschowsky silver staining of the same areas showed the presence of irregular vacuolation and minor axonal damage and loss in SJL mice (c and i) as well as little axonal damage in the PEG_2_ antagonist-treated SJL mice (d and j). Original magnification, a, b, c, d = 50 μm and e, f, g, h, i, j = 100 μm.

To further confirm the inhibition of TMEV-induced demyelinating disease after treatment with AH23848, we compared histopathological evaluations of the brains and spinal cords of the control and AH23848-treated SJL mice at 43 dpi. H&E staining was used to evaluate the evidence of active inflammation and lymphocyte infiltration. LFB staining was used to evaluate axonal demyelination. Bielschowsky silver staining was used to evaluate axonal damage. Lymphocyte infiltration, demyelination and axonal loss were observed in the cerebellum in the brain (Fig 4A and 4C) as well as in the white matter and meninges of the spinal cord (Fig 4E, 4G and 4I) in the control SJL mice. Irregular vacuolation, demyelination and axon loss was also found in the white matter of the spinal cord, the cerebellum and the medulla in the control SJL mice. In contrast, AH23848-treated SJL mice had a high myelin and axon density and less infiltration both in the cerebellum (Fig 4B and 4D) and in the spinal cord (Fig 4F, 4H and 4J). The brains and the spinal cords of the AH23848-treated SJL mice showed near normal histology, and only few histopathological changes were observed. Therefore, the histopathological examinations are consistent with the clinical signs in these mice.

### Reduced production of cytokines in the CNS of AH23848-treated mice compared to control mice

**Fig 5:**
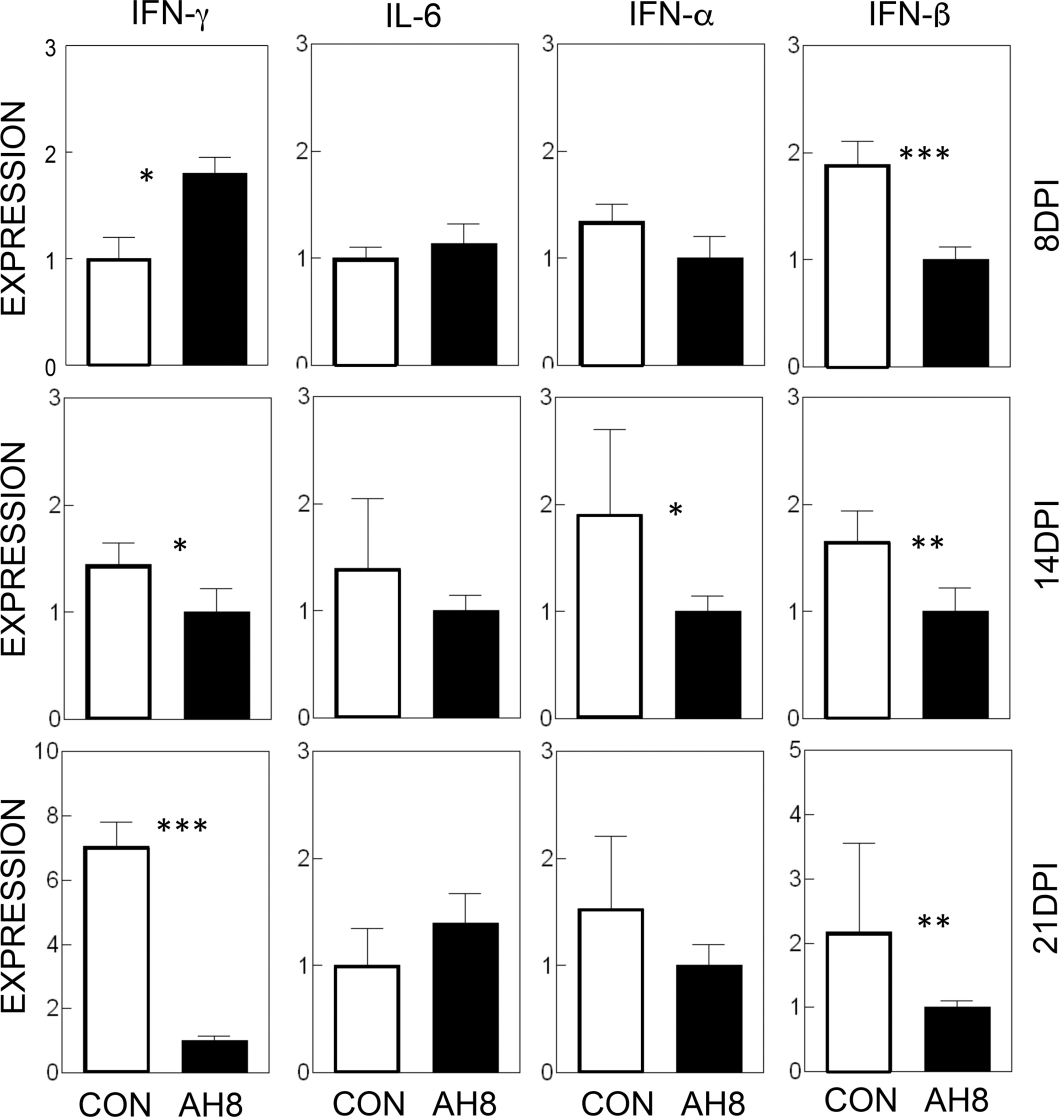
Determination of various inflammatory cytokine levels in the CNS of TMEV-infected mice with or without AH23848 treatment. Cytokine message levels (IFN-γ, IL-6, IFN-α and IFN-β) in the CNS were determined at 8, 14 and 21 dpi using qPCR. Relative values between the control and AH-treated mice were shown. The error bars represent standard deviations (SD) of three samples in each group. The significance of the difference was assessed using two-tailed unpaired Student t test. *, P < 0.05; **, P < 0.01; ***, p < 0.001.

To understand the mechanisms underlying the amelioration of TMEV-IDD by the inhibition of PGE_2_ signaling, we assessed the message levels of several cytokines in the CNS during the development of TMEV-induced demyelinating disease (Fig 5). The results indicate that the initial level of IFN-γ was slightly higher (*p*<0.05) at 8 dpi in AH23848-treated mice compared to control mice. However, the IFN-γ levels became significantly lower (*p*<0.05 and *p*<0.001, respectively) in AH23848-treated mice at 14 and 21 dpi. This could be consequential to the decrease in the viral loads in the CNS of treated mice observed earlier (Fig 3). No significant differences in the levels of IL-6 were observed in these mice at any time point. Only a transiently lower level of IFN-α was observed at 14 dpi (*p*<0.05) in the AH23848-treated mice. However, the levels of IFN-β in the CNS were consistently lower (*p*<0.01) in the treated mice throughout the viral infection. Because very low levels of these cytokines were expressed in uninfected naive mice or mice at early viral infections 0–3 dpi [26], the early time points were not included. These results suggest that the blockade of PGE_2_ signaling may alter the production of IFN-β, which is also known to play a protective or pathogenic role in TMEV-induced demyelinating disease [35, 44].

### Higher proportion of IFN-γ-producing CD4^+^ and CD8^+^ T cells in the CNS of AH23848-treated mice at 8 days post-viral infection

**Fig 6:**
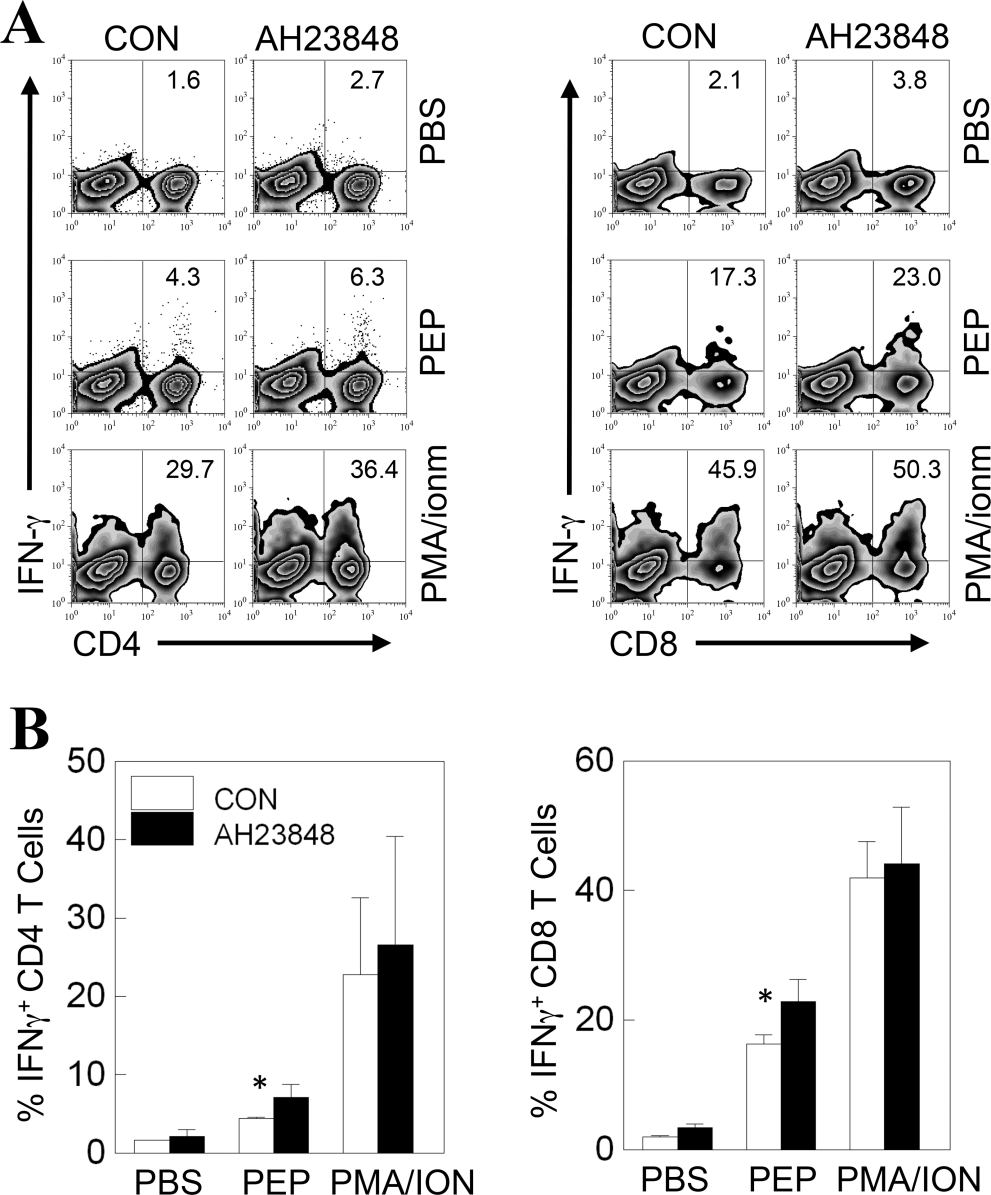
Determination of IFN-γ production by CNS-infiltrating CD4^+^ and CD8^+^ T cells from TMEV-infected mice with or without AH23848 treatment at 8 dpi. **A.** CNS-infiltrating mononuclear cells were restimulated for 6 h with PBS, TMEV epitope peptides or PMA/ionomycin. For CD4^+^ T cells, PEP represents a mixture of 1 μM VP1_233-250_, VP2_74-86_, and VP3_24-37_. For CD8^+^ T cells, PEP represents a mixture of 2 μM VP3_159-166_ and VP3_173-181_. The cells were then stained for CD4 or CD8 and intracellular IFN-γ. The percentages of CD4^+^ or CD8^+^ and IFN-γ^+^ cells are shown in the upper right corner. The data are representative of three independent experiments. **B.** Three separate experimental values were combined and presented as bar graphs. *, P < 0.05.

It has previously been reported that PGE_2_ signaling inhibits the production of IL-12 and IFN-γ. To further assess the potential effects of AH23848 treatment on the responses of IFN-γ-producing CD4^+^ and CD8^+^ T cells in the CNS, we analyzed intracellular IFN-γ production by the T cells 8–9 days post-infection (Fig 6). The AH23848-treated mice showed consistently higher proportion of IFN-γ-producing CD4^+^ (*p*<0.05) and CD8^+^ T cells (*p*<0.05) in response to viral epitopes but not in response to pan-T-cell stimulation. Therefore, greater numbers of Th1 and cytotoxic T cells producing IFN-γ in AH23848-treated mice may have contributed to early viral clearance as these T cell types are critically important in protection against the pathogenesis of demyelinating disease [26, 45, 46].

### Elevated activation of macrophages/microglia in AH23848-treated mice at 8 dpi

**Fig 7:**
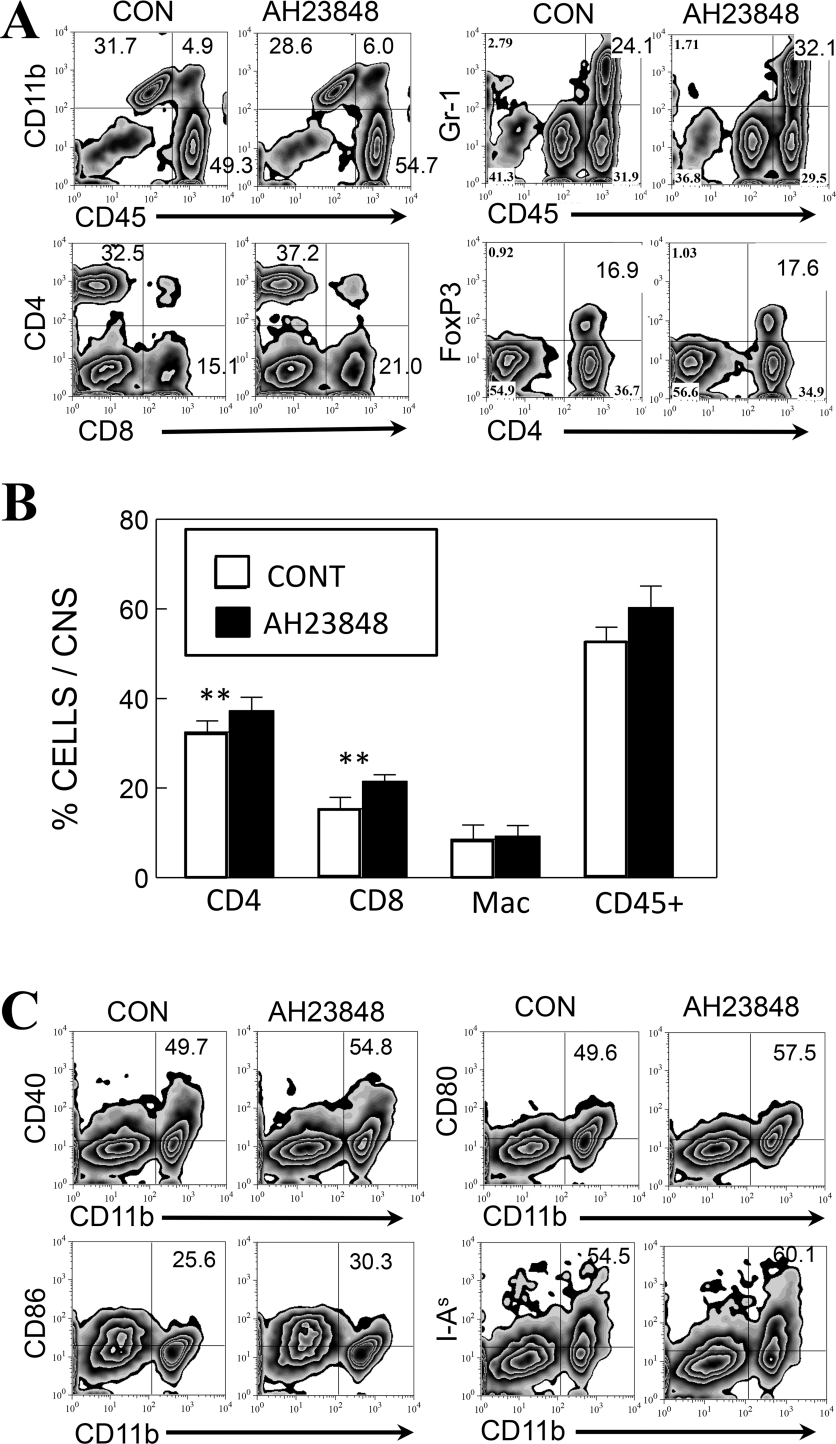
Flow cytometry profiles of CD11b^+^, CD4^+^ and CD8^+^ cells in the CNS of TMEV-infected control and AH23848-treated mice (n = 3) at 8 dpi. **A.** The proportions of CNS-infiltrating CD4^+^ and CD8^+^ cells, CD11b^+^ or Gr-1^+^ cells in conjunction with CD45^+^ and FoxP3^+^ CD4^+^ cells in TMEV-infected control and AH23848-treated SJL mice were compared. **B.** The proportions of CD4^+^ and CD8^+^ cells, CD11b^+^CD45^+^ macrophages and activated CD45^+^ lymphocytes infiltrating the CNS from 3–4 experiments were analyzed using a paired Student’s t test. **, P < 0.01. **C.** Co-stimulatory molecules (CD80, CD86, CD40, I-A^s^) involved in T cell activation were also slightly elevated in infiltrating macrophages from AH23848-treated mice compared to those from control mice, consistent with the elevated IFN-γ-producing T cells in the CNS. Flow cytometry data presented in **A** and **C** are representatives of three independent experiments.

To understand the mechanisms underlying the increased levels of Th1 and CD8^+^ T cell responses producing IFN-γ, we assessed the proportions of CD4^+^ and CD8^+^ T cells as well as CD11b^+^ macrophages/microglia in the CNS in the early stage of viral infection, at 8 dpi (Fig 7). The results suggest that CD11b^+^ macrophages (CD45^hi^) and CD11b^+^ microglia (CD45^low^) were slightly increased in the CNS of virus-infected AH23848-treated mice compared to the CNS of infected control mice. In addition, more infiltration of CD4^+^ and CD8^+^ T cells was observed in the AH23848-treated mice. However, the levels of FoxP3^+^ CD4^+^ T cells were indifferent. The increases in the T cell populations in the AH23848-treated mice compared to the control mice were small but very consistent, and paired t tests analyzing the results of three experiments showed significant differences (*p*<0.01) in the proportion of both infiltrating CD4^+^ and CD8^+^ T cells (Fig 7B). In addition, markers that are associated with antigen presentation to T cells on CD45^+^CD11b^+^ macrophages/microglia in the CNS (CD40, CD80, CD86 and I-A^s^) were also consistently elevated in the AH23848-treated mice (Fig 7C). These results are consistent with the elevated T cell activation in the CNS of AH23848-treated mice compared to the control mice.

### Higher levels of IFN-γ-producing peripheral T cells in AH23848-treated mice

**Fig 8:**
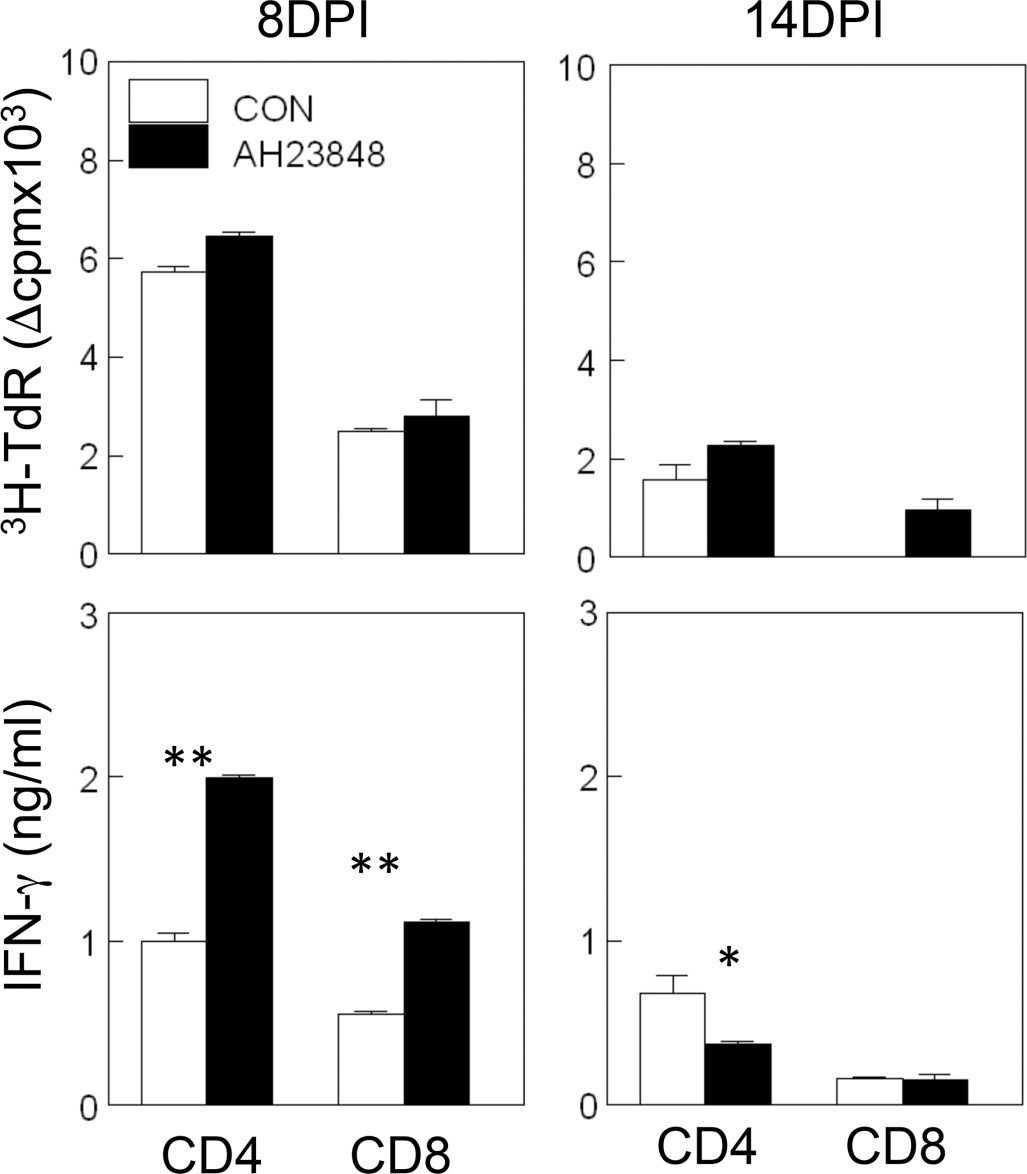
Determination of CD4^+^ and CD8^+^ T cell recall responses in the periphery of TMEV-infected mice with or without AH23848 treatment at 8 and 14 dpi. Proliferative responses to viral epitope peptides for CD4^+^ T cells (1 μM VP1_233-250_, VP2_74-86_, and VP3_24-37_) and those for CD8^+^ T cells (2 μM VP3_159-166_ and VP3_173-181_) were similar between splenic T cells from the AH23848-treated and control mice at both 8 and 14 dpi. However, the production of IFN-γ by the CD4^+^ and CD8^+^ T cells from AH23848-treated mice at 8 dpi was significantly higher than that by the T cells from the control mice. In contrast, CD4^+^ T cells from AH23848-treated mice at 14 dpi produced significantly lower levels of IFN-γ compared to CD4^+^ T cells from control mice.

To further compare the T cell responses in the CNS with those in the periphery, we evaluated the proliferation and IFN-γ production of splenic T cells from mice infected with TMEV at 8 and 14 dpi upon restimulation with viral epitopes (Fig 8). There were no significant differences in the proliferative responses of the CD4^+^ and CD8^+^ T cells between the AH23848-treated and the control mice at both 8 and 14 dpi. In contrast, the levels of IFN-γ production by the CD4^+^ and CD8^+^ T cells at 8 dpi were significantly higher (*p*<0.01 for both CD4^+^ and CD8^+^ T cells) in the AH23848-treated mice than in the control mice. However, their levels of IFN-γ production of CD4^+^ T cells at 14 dpi were significantly lower (*p*<0.05) than in the control mice. Therefore, the cytokine production of splenic T cells appears to be correspond well to those in the CNS; i.e., IFN-γ production in the CNS was slightly elevated at 8 dpi, while the increase is more pronounced in the periphery. Similarly, the production of IFN-γ in the CNS of AH23848-treated mice became significantly lower than the control at 21 dpi, but the production of IFN-γ in the periphery was already lower at 14 dpi. The reduction in IFN-γ production may reflect the decreased viral load levels in the CNS vs. the periphery.

## Discussion

Many viral infections stimulate TLR- or TNF-α-mediated signaling, resulting in the activation of the NLRP3 inflammasome and leading to the subsequent production of IL-1β and COX-2 [20, 21]. TMEV infection induces vigorous innate immune responses, including type I IFNs, various chemokines such as CXCL-1 and cytokines such as IL-6 [37, 47]. The innate immune responses following TMEV infection are induced by various pattern recognition receptors, including TLR2, TLR3, MDA-5 and PKR [1–3, 48]. Activation of the NLRP3 inflammasome requires stimulation via a TLR and the activation of caspase-1 associated with the cleavage of pro-IL-1β and pro-IL-18 [20]. We have also reported that TLR3, MDA-5, IL-1β and type I IFNs play critical roles in the development of TMEV-induced demyelinating disease [3, 9, 44, 49]. The presence of TLRs, IL-1β or type I IFNs is necessary for protection from the development of TMEV-induced demyelinating disease, yet excessive signaling through these molecules promotes the development of the disease. Therefore, it is expected that activation of the NLRP3 inflammasomes and its downstream signaling likely affect the outcome of the development of viral demyelinating disease.

In this study, we assessed the levels of NLRP3 inflammasome activation and its downstream signaling pathways. NLRP3 expression was significantly upregulated after infection with TMEV in all cell types tested (i.e., bone marrow-derived dendritic cells, neonatal brain cells, and neonatal oligodendrocytes) derived from susceptible SJL mice but not in cells derived from resistant B6 mice (Fig 1). The NLRP3 inflammasome is most likely activated via TLR3, TLR2 and/or MDA5 upon TMEV infection because various critical innate immune responses, including IL-1β, are activated by these receptors [1–3]. Furthermore, our previous studies indicated that IL-1β, a downstream product of NLRP3 activation, plays an important role in the protection and the pathogenesis of demyelinating disease depending on the level and time after viral infection [9].

Many viral infections induce COX-2 and PGE_2_, which affects the pathogenesis of many different viruses [21]. Oligodendrocytes from TMEV-infected mice expressed COX-2, and inhibition of COX-2 limited the development of TMEV-induced demyelinating disease [22]. The investigators attributed the COX-2 dependent pathogenesis of demyelinating disease to Cox-2-associated oligodendrocyte death. Our results indicated that TMEV infection caused significant increases in the levels of COX-2 and mPGES-1 mRNAs in glial cells from SJL mice (Fig 2). These results are consistent with the previous report that the levels of COX-2 and PGE_2_ are increased after infection of astrocytes, oligodendrocytes and brain endothelial cells with TMEV [22, 50, 51]. In contrast, only minimal increases were found in glial cells from B6 mice (Fig 2), suggesting that the levels of NLRP3 activation (Fig 1) and its subsequent downstream signaling are particularly elevated in the glial cells of susceptible mice, but not in those of resistant mice, after TMEV infection. Although the levels of COX-2 mRNA in infected DCs from SJL and B6 mice were both similarly elevated, no significant induction of mPGES-1 was observed in cells from B6 mice. These results demonstrated that the expression of the two major downstream components of NLRP3, COX-2 and mPGES-1, is significantly elevated in cells from susceptible mice compared to the lack of activation or only partial activation in cells from resistant mice. Inhibition of Enterovirus 71-induced COX-2 expression and PGE_2_ production had an antiviral effect on some members of Picornaviridae [52]. Therefore, it is likely that COX-2 and/or PGE_2_ contribute to the inhibition of anti-viral immune responses.

mPGES-2 is constitutively expressed in neurons and is induced in some glial cells, such as activated microglia [39]. Notably, PGES-2 was significantly elevated only in astrocytes, but not in oligodendrocytes and microglia, after TMEV infection, suggesting that different glial cell types respond differently to the activation of PGES-2. Although PGDS plays a role in host immunity [53] and is expressed in microglia [40], it does not appear to participate in the development of TMEV-induced demyelinating disease because PGDS levels were not altered following TMEV infection (Fig 2).

A reduced level of IL-6 and a reduced number of T cells was previously shown in mice lacking COX-2 or EP4 [42]. Because the levels of IL-6 and T cells in the CNS are known to play important roles in the pathogenesis of TMEV-induced demyelinating disease [26, 27, 54–56], the signals downstream of the NLRP3 inflammasome are likely to be involved in the pathogenesis of TMEV-induced, immune-mediated demyelinating disease. To further determine whether PGE_2_ signaling affects the pathogenesis of TMEV-induced demyelinating disease, PGE_2_-EP4 signaling was blocked in mice during TMEV infection using AH23848 (Fig 3). The use of the EP4 antagonist AH23848 was previously well established [31, 32, 57, 58]. Disease progression was significantly slower and incidence was reduced in mice treated with AH23848 compared to the control mice, accompanied by decreased viral loads in the CNS (Figs 3 & 4). These results are consistent with the effects of a different EP4 antagonist, ONO-AE1-329, at EAE onset [24, 41], which included delayed disease progression and reduced permeability of the blood-brain barrier. In addition, the pathogenic effects of PGE2 signaling on the development of EAE were previously well documented [41, 59]. Therefore, the effects of PGE_2_ blockade on the development of immune-mediated neuroinflammatory diseases are common in these viral and autoimmune models of MS. However, AH23848 is dual antagonist of thromboxane A2 (TXA2) and PGE2 receptors, which are products of constitutive COX-1 and inducible COX-2, respectively [60, 61]. The potential inhibition of TXA2 synthesis on the pathogenesis of TMEV-induced demyelinating disease is unknown at this time. Nevertheless, the inhibition of TXA2 level by AH23848 may also contribute to lower cellular immune responses and inflammatory tissue injury [62, 63].

The underlying mechanisms of the PGE_2_-mediated enhancement of viral pathogenesis are not yet clear. The levels of various cytokines involved in innate and adaptive immunity were altered in AH23848-treated TMEV-infected mice (Fig 5). PGE_2_ regulates T-cell activation and differentiation positively or negatively depending on the microenvironment of the cell, the maturation and activation state of the cell, the type of EP receptor involved, and the local concentration of PGE_2_ [64]. PGE_2_ acts as a potent pro-inflammatory mediator by inducing IL-8 gene transcription in activated T cells [65]. However, PGE_2_ prevents CD95L upregulation in T cells in response to TCR/CD3 stimulation, thereby avoiding activated T cell killing of target macrophages [66]. The level of IFN-γ was slightly higher at 8 dpi but became significantly lower at 21 dpi in the AH23848-treated mice. The early elevation of IFN-γ production reflecting the abundant protective T cells including CD8^+^ cytotoxic T cells and Th1 cells is critically important in the protection of mice from the pathogenesis of TMEV-induced demyelinating disease [33, 67]. Thereby AH23848-treated mice may display more efficient elimination of the initial viral loads. In contrast, the levels of type I IFNs were lower throughout in the treated mice. Excessive levels of type I IFNs in the susceptible SJL mice are known to be a potent inhibitory factor against the protective immune responses to TMEV by inhibiting the maturation and promoting the apoptosis of antigen-presenting cells [35, 44]. Therefore, the lower levels of type I IFNs in the AH23848-treated mice may permit the generation of elevated IFN-γ producing T cells (Fig 6) in combination with higher infiltration of more functional cells into the CNS (Fig 7). The initial high level of IFN-γ and the consequent reduction of viral loads by the elevated protective IFN-γ-producing T cell responses in the AH23848-treated mice (Fig 3) may inhibit the production of excessive type I interferons (IFNs).

Because mice lacking COX-2 or EP4 display a reduced level of IL-6 [42], a reduction in the level of IL-6 in AH-23848-treated mice was expected. However, the IL-6 level was not altered compared to the control mice (Fig 5). It is possible that the production of IL-6 in TMEV-infected mice may not be entirely dependent on NLRP3 inflammasome signals. For example, non-TLR-mediated signaling components, such as PKR and other pattern recognition receptors (e.g., MDA-5), may predominantly participate in the production of IL-6. Similarly, it has been shown that FoxP3^+^ cells are preferentially induced under high PGE_2_ [64, 68]. FoxP3^+^ regulatory cells are known to play an important role in the pathogenesis of TMEV-induced demyelinating disease [69]. However, the level of FoxP3^+^ cells in AH23848-treated mice was similar to that of control mice (Fig 7). Therefore, the contribution of the NLRP3 inflammasome and downstream PGE_2_ in the generation of FoxP3 regulatory cells appears to be minimal, perhaps reflecting the redundancy of signals for FoxP3 generation. Taken together, these results strongly suggest that the inhibition of PGE_2_ signaling would be a very important tool for controlling the pathogenesis of virally induced chronic inflammatory diseases.

## Supporting information

S1 Table Clinical scores of individual mice during the course of TMEV infection. (DOCX)

## Abbreviations

PGE_2_: prostaglandin E2
CNS: central nervous system
NLRP3: node-like receptor protein 3
dpi: days post-infection
MS: Multiple sclerosis
CNS: central nervous system
TMEV: Theiler’s murine encephalomyelitis virus
TMEV-IDD: TMEV-induced demyelinating disease
PCR: polymerase chain reaction
MOI: multiplicity of infection
PFU: plaque-forming unit
PI: post-infection
S mix: peptide mix derived from Structural proteins
NS mix: peptide mix derived from Non-structural proteins
