# Supplementary material for: Prostaglandin E2 produced following infection with Theiler's virus promotes the pathogenesis of demyelinating disease
Source: PLoS One. 2017 Apr 26;12(4):e0176406. doi: 10.1371/journal.pone.0176406 (PMC5406002; doi:10.1371/journal.pone.0176406)
Supplement: S1 Table — (DOCX) [file pone.0176406.s001.docx]

Table 1: Clinical Scores of individual mice and the means and SD.

Clinical Scores at the following dpi

| Control |  |  |  |  |  |  |  |  |
| --- | --- | --- | --- | --- | --- | --- | --- | --- |
| Group | 13 | 21 | 28 | 34 | 42 | 49 | 56 | dpi |
| 1 | 1.5 | 2 | 2.5 | 3 | 4 | 4 | 4 |  |
| 2 | 0 | 1 | 1 | 2 | 3 | 3 | 3 |  |
| 3 | 0 | 0.5 | 1 | 1 | 5 | 5 | 5 |  |
| 4 | 0 | 0.5 | 0.5 | 0.5 | 2 | 2.5 | 2.5 |  |
| 5 | 0 | 0.5 | 0.5 | 0.5 | 0.5 | 2 | 2.5 |  |
| 6 | 0 | 0 | 0 | 0.5 | 0.5 | 1 | 1 |  |
| 7 | 0 | 1 | 2 | 2.5 | 4 | 4.5 | 4.5 |  |
| 8 | 0 | 0 | 1 | 1.5 | 3.5 | 3.5 | 3.5 |  |
| 9 | 0 | 0 | 0.5 | 1.5 | 3.5 | 3.5 | 3.5 |  |
| Mean | 0.167 | 0.611 | 1.000 | 1.444 | 2.89 | 3.222 | 3.278 |  |
| SD | 0.5 | 0.651 | 0.791 | 0.917 | 1.577 | 1.252 | 1.202 |  |
| Affected | 11.11 | 66.67 | 88.89 | 100 | 100 | 100 | 100 | % |

Clinical Scores at the following dpi

| AH23848 |  |  |  |  |  |  |  |  |
| --- | --- | --- | --- | --- | --- | --- | --- | --- |
| Group | 13 | 21 | 28 | 34 | 42 | 49 | 56 | dpi |
| 1 | 0 | 0 | 0.5 | 1 | 1 | 1 | 1 |  |
| 2 | 0 | 0 | 0 | 0 | 0 | 0 | 0 |  |
| 3 | 0 | 0 | 0 | 0 | 0 | 0 | 0 |  |
| 4 | 0 | 0 | 0 | 0 | 0 | 0 | 0 |  |
| 5 | 0 | 0 | 0 | 0 | 0 | 0 | 0 |  |
| 6 | 0 | 0 | 1 | 1.5 | 2 | 2.5 | 3 |  |
| 7 | 0 | 0 | 1 | 2 | 3 | 3 | 3 |  |
| 8 | 0 | 0 | 0.5 | 1 | 2 | 2.5 | 2.5 |  |
| 9 | 0 | 0 | 0 | 0 | 0.5 | 1.5 | 2 |  |
| 10 | 0 | 0 | 0 | 0 | 0 | 0 | 0 |  |
| Mean | 0 | 0 | 0.3 | 0.55 | 0.85 | 1.05 | 1.15 |  |
| SD | 0 | 0 | 0.422 | 0.762 | 1.107 | 1.235 | 1.334 |  |
| Affected | 0 | 0 | 40 | 40 | 70 | 70 | 70 | % |
